# Association between early coagulation disorders and the risk of severe acute kidney injury in traumatic brain injury patients: a retrospective cohort study using the MIMIC-IV database

**DOI:** 10.3389/fneur.2024.1407107

**Published:** 2025-02-18

**Authors:** Yu Gao, Yong Li, Hai Zhou, Xin Wang, Guojun Wang, Lin Zhu

**Affiliations:** ^1^Department of Critical Care Medicine, Binhai County People’s Hospital, Affiliated Binhai Hospital, Kangda College of Nanjing Medical University, Yancheng, Jiangsu, China; ^2^Department of Neurosurgery, Binhai County People’s Hospital, Affiliated Binhai Hospital, Kangda College of Nanjing Medical University, Yancheng, Jiangsu, China; ^3^Department of Traditional Chinese Medicine, Binhai County People’s Hospital, Affiliated Binhai Hospital, Kangda College of Nanjing Medical University, Yancheng, Jiangsu, China

**Keywords:** early coagulation disorder, acute kidney injury, traumatic brain injury, MIMIC-IV database, retrospective cohort study

## Abstract

**Aim:**

Acute kidney injury (AKI) and coagulation disorders are two common complications of traumatic brain injury (TBI) that are associated with poor prognosis. However, the relationship between early coagulation disorders and the risk of severe AKI in TBI patients remains unclear. This study aimed to explore the association between early coagulation disorders and the risk of severe AKI in TBI patients admitted to the intensive care unit (ICU).

**Methods:**

In this retrospective cohort study, adults diagnosed with TBI were selected from the Medical Information Mart for Intensive Care (MIMIC)-IV database. The outcome was the risk of severe AKI within 7 days of ICU admission in TBI patients. Covariates including sociodemographic information, vital signs, scoring systems, and laboratory parameters were extracted from the database. Univariable and multivariable Cox proportional hazard regression models were used to assess the association between early coagulation disorders and the risk of severe AKI within 7 days of admission to the ICU in TBI patients. Subgroup analyses based on age and the Glasgow Coma Scale (GCS) score were further conducted to assess the association.

**Results:**

A total of 846 patients were finally included, of whom 187 (22.10%) had severe AKI. After adjusting for all covariates, the TBI patients with early coagulation disorders had a higher risk of developing severe AKI within 7 days of ICU admission compared to the TBI patients without early coagulation disorders (hazard ratio (HR) = 1.40, 95% confidence interval (CI): 1.04–1.89), particularly among those aged ≥65 years (HR = 1.46, 95%CI: 1.01–2.04) and those with a GCS score ≤ 13 (HR = 1.91, 95%CI: 1.16–3.15).

**Conclusion:**

TBI patients with early coagulation disorders had a higher risk of developing severe AKI within 7 days of ICU admission. This may serve as a promising biomarker and could be helpful for managing kidney health in TBI patients.

## Introduction

Traumatic brain injury (TBI) is caused by a bump, blow, or jolt to the head, or by a penetrating head injury that disrupts the normal functions of the brain (1). While TBI often results from sudden trauma to the head its deleterious effects on patients can be lifelong and dynamic (2). With approximately 70 million people experiencing TBI each year worldwide, it remains a growing public health concern and the leading cause of death and long-term disability among all trauma-related injuries (3, 4). The high mortality rate associated with TBI largely depends on the systemic complications of secondary brain injury. Acute kidney injury (AKI), which is characterized by a rapid loss of excretory function, is a common complication following TBI (5). The incidence of AKI in patients with both TBI and cerebrovascular disease is approximately 10%, which may lead to a longer hospital stay and a poorer prognosis (5, 6). Therefore, the identification of biomarkers related to the risk of severe AKI in TBI patients is of great significance for the management of kidney health in TBI patients admitted to the intensive care unit (ICU).

Coagulation disorders refer to blood coagulation abnormalities caused by congenital or acquired deficiencies in coagulation factors, vascular wall damage, or platelet dysfunction (7). Coagulation disorders often occur after TBI, and the incidence of TBI-related coagulopathy is reported to be 10–87.5% (8). A massive release of tissue factor, altered protein C homeostasis, and platelet hyperactivity in TBI patients may lead to the development of TBI-related coagulation disorders (8, 9). Mechanically, the activation of the coagulopathy system and the downregulation of the anticoagulant pathway result in high levels of thrombin expression and increased fibrin deposition in the microcirculation (10). Previous studies have reported that elevated levels of coagulation markers, such as the international normalized ratio (INR) and activated partial thromboplastin time (APTT), are associated with a higher risk of AKI in patients experiencing septic shock. Additionally, these elevated levels are linked to an increased risk of in-hospital death in TBI patients (8, 11). However, less is known about the association between early coagulation disorders and the risk of severe AKI in TBI patients. Herein, we speculated that early coagulation disorders may be a risk factor for severe AKI in TBI patients admitted to the ICU.

The present study aimed to explore the relationship between early coagulation disorders and the risk of severe AKI in TBI patients admitted to the ICU, using data from the Medical Information Mart for Intensive Care IV (MIMIC-IV) database. Subgroup analyses based on age and the Glasgow Coma Scale (GCS) score were conducted to evaluate the association in different subpopulations.

## Methods

### Study design and participants

We conducted a retrospective cohort study utilizing the MIMIC-IV database. The MIMIC-IV is a large, open, and freely accessible database, with the latest version being MIMIC-IV version 1.0.^1^ This database contains comprehensive information about approximately 2,50,000 patients who were hospitalized from 2008 to 2019, providing robust data support for clinical studies (12). The database was approved by the Massachusetts Institute of Technology and the Beth Israel Deaconess Medical Center, and consent was obtained for the collection of the original data. Each patient’s length of stay, laboratory tests, medication treatment, vital signs, and other comprehensive data were recorded. Moreover, the MIMIC-IV database anonymizes patient information, so informed consent was not required.

A total of 2,382 TBI patients aged ≥18 years and admitted to the ICU were initially screened from the MIMIC-IV database. Patients who were admitted to the ICU for less than 24 h, diagnosed with AKI or end-stage renal disease upon ICU admission, or lacked important data on platelet (PLT) count, activated partial thromboplastin time (APTT), international normalized ratio (INR), and GCS score were excluded from the study. Finally, 846 eligible TBI patients were included for further analysis.

### AKI and severe AKI definitions

The primary outcome of our study was the risk of severe AKI within 7 days of ICU admission. The Kidney Disease: Improving Global Outcomes (KDIGO) criteria were used to determine the occurrence of AKI. According to this definition, stage 1 is diagnosed when there is an increase in serum creatinine (Scr) to 1.5 times the baseline value or urine output (UO) <0.5 mL/kg/h for 6–12 h (13). Stage 2 is defined by an increase in Scr of 2.0–2.9 times the baseline level or UO <0.5 mL/kg/h for ≥12 h. Stage 3 is defined as an increase in Scr of 3.0 times the baseline value or UO <0.3 mL/kg/h for ≥24 h or anuria for ≥12 h. According to KDIGO, stages 2 and 3 are considered severe AKI (14).

### Outcome definition and follow-up

The endpoint of the present study was the risk of severe AKI within 7 days of ICU admission in TBI patients. Follow-up began at the time of the patient’s first ICU admission and ended when severe AKI occurred or 7 days after the ICU admission.

### Definition of early coagulation disorders

PLT, INR, and APTT scores were used to define early coagulation disorders. PLT scores >150 × 10^9^/L, 100–150 × 10^9^/L, and < 100 × 10^9^/L were assigned 0, 1, and 2, respectively. INR scores <1.2, 1.2–1.4, and > 1.4 were assigned 0, 1, and 2, respectively. APTT scores <37 s, 37–39 s, and > 39 s were assigned 0, 1, and 2, respectively. A total score greater than 0 was considered indicative of an early coagulation disorder (15).

### Potential covariates

The variables such as demographics, vital signs, laboratory tests, treatment and medication history, scoring systems, and other variables were extracted. The demographic and vital sign variables included age, sex, ethnicity, marital status, heart rate, systolic blood pressure (SBP), diastolic blood pressure (DBP), respiratory rate, and temperature. The laboratory test variables included oxygen saturation (SpO2), white blood cell (WBC) count, hemoglobin, red blood cell distribution width (RDW), blood urea nitrogen (BUN), glucose, anion gap, calcium, sodium, potassium, chloride, bicarbonate, mannitol, INR, PLT, and APTT. The treatment and medication history variables included platelet transfusion, the use of vasopressors and diuretics, and vitamin K. The scoring systems included the Simplified Acute Physiology Score II (SAPS II), the Charlson Comorbidity Index (CCI), and the GCS. Other collected data included insurance type, ICU type, 24 h UO, the use of mechanical ventilation, neurosurgical interventions, and the time of admission.

### Statistical analysis

All statistical analyses were performed using SAS 9.4 (SAS Institute Inc., Cary, NC, United States). Continuous data were expressed as means and standard errors (SEs), and the comparison between the two groups was conducted using the *t*-test. Qualitative data were expressed as numbers and proportions [n (%)], and the chi-squared (χ^2^) test was used for the comparison between the two groups. Sensitivity analysis was performed to compare whether the results differed before and after the imputation of missing data. The univariable Cox proportional hazard model was utilized to screen the covariates related to severe AKI in TBI patients (Supplementary Table S1). Univariable and multivariable Cox proportional hazard models were used to assess the association between early coagulation disorders and the risk of severe AKI within 7 days of ICU admission in TBI patients, with hazard ratios (HRs) and 95% confidence intervals (CIs). Model 1 was the crude model without adjusting for the covariates. Model 2 was adjusted for SAPS II, calcium, mechanical ventilation, vasopressors, and platelet transfusion. Subgroup analyses were further conducted to explore the association based on age and GCS scores. A two-sided *p*-value of <0.05 was considered statistically significant.

## Results

### Baseline characteristics

**Figure 1:**
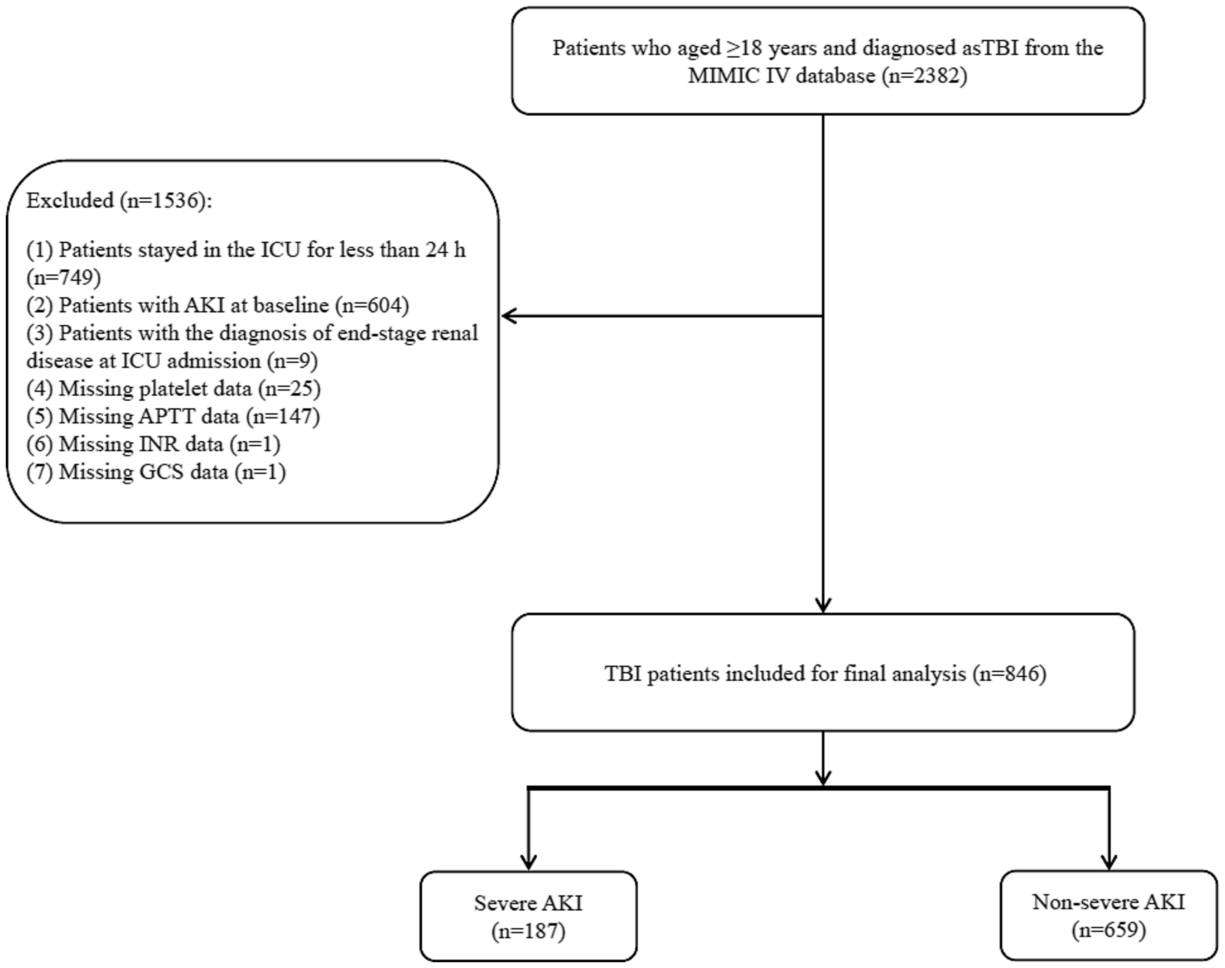
A flowchart of the population screening.

A flowchart of the population screening is shown in Figure 1. A total of 2,382 TBI patients were screened. Among them, 749 patients who were admitted to the ICU for less than 24 h, 604 patients with AKI at baseline, 9 patients who were diagnosed with end-stage kidney disease at the time of hospital admission, 25 patients with missing APTT data, 1 patient with missing INR data, and 1 patient with missing GCS data were excluded. Overall, 846 patients were included, with a mean age of 64.53 (20.31) years, and 62.29% were male. The sensitivity analysis showed that the results before and after the imputation of the missing variables were stable (all *p* > 0.05) (Supplementary Table S2). Table 1 shows the basic demographic characteristics and covariates of the population. The proportion of the highest total score of the PLT, INR, and APTT in the severe AKI group was significantly higher than that in the non-severe AKI group (1.60% vs. 0.15%). Differences were found between the levels of SAPS II, RDW, platelet count, glucose, calcium, mechanical ventilation, the use of vasopressors and diuretics, and platelet transfusion between the severe AKI group and non-severe AKI group (all *p* < 0.05).

**Table 1:** Characteristics of the patients with TBI.

Variables	Total (*n* = 846)	Non-severe AKI (*n* = 659)	Severe AKI (*n* = 187)	Statistics	*p*
Age, years, Mean ± SD	64.53 ± 20.31	63.86 ± 20.51	66.89 ± 19.43	*t* = −1.80	0.072
Sex, *n* (%)				χ^2^ = 0.008	0.930
Female	319 (37.71)	249 (37.78)	70 (37.43)		
Male	527 (62.29)	410 (62.22)	117 (62.57)		
Ethnicity, *n* (%)				χ^2^ = 0.822	0.663
White	534 (63.12)	414 (62.82)	120 (64.17)		
Black	50 (5.91)	37 (5.61)	13 (6.95)		
Other	262 (30.97)	208 (31.56)	54 (28.88)		
Insurance, *n* (%)				χ^2^ = 1.441	0.486
Medicaid	59 (6.97)	46 (6.98)	13 (6.95)		
Medicare	362 (42.79)	275 (41.73)	87 (46.52)		
Other	425 (50.24)	338 (51.29)	87 (46.52)		
Marital status, *n* (%)				χ^2^ = 1.351	0.245
Married	572 (67.61)	439 (66.62)	133 (71.12)		
Spinsterhood	274 (32.39)	220 (33.38)	54 (28.88)		
ICU type, *n* (%)				χ^2^ = 0.346	0.841
SICU	230 (27.19)	176 (26.71)	54 (28.88)		
TSICU	315 (37.23)	247 (37.48)	68 (36.36)		
Other	301 (35.58)	236 (35.81)	65 (34.76)		
24 h urine output, ml, M (Q_1_,Q_3_)	1942.00 (1350.00, 2649.00)	1900.00 (1350.00, 2650.00)	1970.00 (1380.00, 2625.00)	Z = 0.065	0.948
Heart rate, bpm, Mean ± SD	83.89 ± 18.07	83.39 ± 17.69	85.66 ± 19.29	*t* = −1.52	0.129
Systolic, mmHg, Mean ± SD	133.04 ± 21.80	132.61 ± 21.67	134.53 ± 22.23	*t* = −1.07	0.287
Diastolic, mmHg, Mean ± SD	72.91 ± 16.69	72.88 ± 16.83	72.98 ± 16.24	*t* = −0.07	0.942
Respiratory rate, insp/min, Mean ± SD	18.38 ± 5.01	18.33 ± 5.06	18.56 ± 4.84	*t* = −0.54	0.593
Temperature, Deg. C, Mean ± SD	36.84 ± 0.70	36.86 ± 0.66	36.77 ± 0.83	*t* = 1.29	0.197
SPO_2_, %, Mean ± SD	97.61 ± 2.85	97.58 ± 2.83	97.73 ± 2.94	*t* = −0.67	0.505
SAPS II, M (Q_1_,Q_3_)	31.00 (25.00, 38.00)	31.00 (24.00, 37.00)	34.00 (27.00, 42.00)	Z = 3.984	<0.001
GCS, Mean ± SD	13.04 ± 2.51	13.09 ± 2.35	12.85 ± 3.01	*t* = 1.02	0.308
GCS, *n* (%)				χ^2^ = 0.786	0.375
≤13	345 (40.78)	274 (41.58)	71 (37.97)		
14–15	501 (59.22)	385 (58.42)	116 (62.03)		
CCI, M (Q_1_,Q_3_)	1.00 (0.00, 2.00)	1.00 (0.00, 2.00)	1.00 (0.00, 3.00)	Z = 2.982	0.003
WBC, K/uL, M (Q_1_,Q_3_)	9.65 (7.20, 12.70)	9.80 (7.40, 12.80)	9.20 (6.60, 12.70)	Z = −1.152	0.249
Hematocrit, %, Mean ± SD	34.35 ± 5.60	34.53 ± 5.51	33.73 ± 5.86	*t* = 1.71	0.087
Hemoglobin, g/dL, Mean ± SD	11.46 ± 1.96	11.51 ± 1.95	11.28 ± 2.01	*t* = 1.43	0.153
RDW, %, Mean ± SD	14.08 ± 1.87	14.01 ± 1.77	14.36 ± 2.17	*t* = −2.06	0.041
BUN, mg/dL, M (Q_1_,Q_3_)	15.00 (11.00, 20.00)	15.00 (10.00, 20.00)	15.00 (11.00, 21.00)	Z = 1.207	0.227
Glucose, mg/dL, M (Q_1_,Q_3_)	119.50 (101.00, 147.00)	118.00 (100.00, 143.00)	127.00 (103.00, 151.00)	Z = 2.597	0.009
Calcium, mg/dL, M (Q_1_,Q_3_)	8.60 (7.90, 9.00)	8.60 (8.00, 9.00)	8.40 (7.70, 8.90)	Z = −2.463	0.014
Sodium, mEq/L, Mean ± SD	139.02 ± 4.79	139.20 ± 4.62	138.41 ± 5.32	*t* = 1.85	0.066
Potassium, mEq/L, Mean ± SD	4.03 ± 0.64	4.05 ± 0.65	3.97 ± 0.60	*t* = 1.54	0.125
Chloride, mEq/L, Mean ± SD	103.66 ± 5.41	103.72 ± 5.17	103.44 ± 6.16	*t* = 0.55	0.580
Bicarbonate, mEq/L, Mean ± SD	23.33 ± 3.63	23.31 ± 3.51	23.40 ± 4.04	*t* = −0.26	0.791
Anion gap, mEq/L, Mean ± SD	15.00 ± 3.53	15.06 ± 3.62	14.77 ± 3.21	*t* = 1.07	0.285
Mechanical ventilation, *n* (%)				χ^2^ = 11.537	<0.001
No	335 (39.60)	281 (42.64)	54 (28.88)		
Yes	511 (60.40)	378 (57.36)	133 (71.12)		
Vasopressors, *n* (%)				χ^2^ = 10.537	0.001
No	763 (90.19)	606 (91.96)	157 (83.96)		
Yes	83 (9.81)	53 (8.04)	30 (16.04)		
Platelet transfusion, *n* (%)				χ^2^ = 14.614	<0.001
No	746 (88.18)	596 (90.44)	150 (80.21)		
Yes	100 (11.82)	63 (9.56)	37 (19.79)		
Mannitol, *n* (%)				–	0.090
No	828 (97.87)	648 (98.33)	180 (96.26)		
Yes	18 (2.13)	11 (1.67)	7 (3.74)		
Neurosurgical intervention, *n* (%)				–	0.654
No	839 (99.17)	654 (99.24)	185 (98.93)		
Yes	7 (0.83)	5 (0.76)	2 (1.07)		
Diuretic, *n* (%)				χ^2^ = 4.796	0.029
No	811 (95.86)	637 (96.66)	174 (93.05)		
Yes	35 (4.14)	22 (3.34)	13 (6.95)		
Vitamin K, *n* (%)				–	0.785
No	826 (97.64)	644 (97.72)	182 (97.33)		
Yes	20 (2.36)	15 (2.28)	5 (2.67)		
Time, days, M (Q_1_,Q_3_)	4.23 (2.30, 6.86)	4.96 (3.05, 7.00)	2.04 (1.49, 3.46)	Z = −13.010	<0.001
INR, Mean ± SD	1.20 ± 0.25	1.20 ± 0.25	1.23 ± 0.23	*t* = −1.93	0.054
Platelet, K/uL, M (Q_1_,Q_3_)	187.50 (147.00, 242.00)	190.00 (152.00, 242.00)	170.00 (127.00, 235.00)	Z = −2.708	0.007
APTT, sec, Mean ± SD	28.90 ± 8.44	28.80 ± 8.75	29.25 ± 7.26	*t* = −0.71	0.479
Platelet score, *n* (%)				χ^2^ = 19.102	<0.001
0	621 (73.40)	507 (76.93)	114 (60.96)		
1	158 (18.68)	106 (16.08)	52 (27.81)		
2	67 (7.92)	46 (6.98)	21 (11.23)		
INR score, *n* (%)				χ^2^ = 6.068	0.048
0	616 (72.81)	493 (74.81)	123 (65.78)		
1	150 (17.73)	109 (16.54)	41 (21.93)		
2	80 (9.46)	57 (8.65)	23 (12.30)		
APTT score, *n* (%)				χ^2^ = 4.182	0.124
0	810 (95.74)	636 (96.51)	174 (93.05)		
1	9 (1.06)	5 (0.76)	4 (2.14)		
2	27 (3.19)	18 (2.73)	9 (4.81)		
Total score, M (Q_1_,Q_3_)	0.00 (0.00, 1.00)	0.00 (0.00, 1.00)	1.00 (0.00, 2.00)	Z = 4.070	<0.001
Total score, *n* (%)				–	<0.001
0	483 (57.09)	400 (60.70)	83 (44.39)		
1	184 (21.75)	133 (20.18)	51 (27.27)		
2	102 (12.06)	76 (11.53)	26 (13.90)		
3	46 (5.44)	29 (4.40)	17 (9.09)		
4	20 (2.36)	16 (2.43)	4 (2.14)		
5	7 (0.83)	4 (0.61)	3 (1.60)		
6	4 (0.47)	1 (0.15)	3 (1.60)		
Early Coagulation Disorder, *n* (%)				χ^2^ = 15.824	<0.001
No	483 (57.09)	400 (60.70)	83 (44.39)		
Yes	363 (42.91)	259 (39.30)	104 (55.61)		

*t, t-test; Z, Z-test; χ2, chi-square; AKI, acute kidney disease; SICU, surgical intensive care unit; TSICU, trauma SICU; SAPS II, Simplified Acute Physiology Score II; GCS, Glasgow Coma Scale; WBC, white blood cell; BUN, blood urea nitrogen; RDW, red blood cell distribution width; INR, international normalized ratio; APTT, activated partial thromboplastin time; SD, standard deviation, M, median, Q1, 1st quartile, Q3, 3st quartile.*

### Association between early coagulation disorders and severe AKI

**Figure 2:**
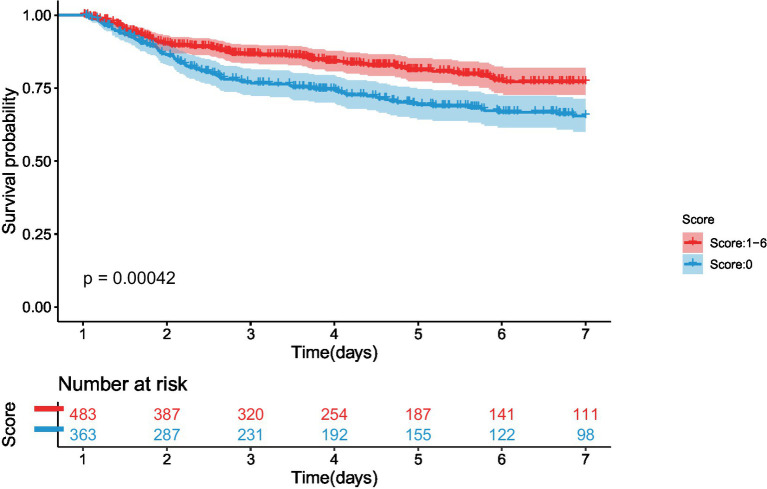
The association between early coagulation disorders and the risk of severe AKI in the patients with TBI admitted to the ICU.

We employed two Cox proportional hazard regression models to investigate the association between early coagulation disorders and the risk of severe AKI in TBI patients admitted to the ICU, as presented in Table 2. In model 2, which was adjusted for SAPSII, calcium, mechanical ventilation, the use of vasopressors, and platelet transfusion, we observed that the patients with TBI and early coagulation disorders had a 40% increased risk of severe AKI compared to the patients without early coagulation disorders (HR = 1.40, 95%CI: 1.04–1.89) (*p* < 0.05). In addition, the restricted cubic spline (RCS) demonstrated a positive linear correlation between early coagulation disorders and the risk of severe AKI in TBI patients admitted to the ICU (*P*_overall_ = 0.0005; *P*_nonlinearity_ = 0.4349). The Kaplan–Meier curve also showed that the patients with TBI and early coagulation disorders had a higher risk of severe AKI compared to those without early coagulation disorders (*p* = 0.00042) (Figure 2).

**Table 2:** Association between early coagulation disorders and the risk of AKI in the patients with TBI admitted to the ICU.

Variables	Model 1		Model 2	
	HR (95%CI)	*p*	HR (95%CI)	*p*
Early coagulation disorder				
No	Ref		Ref	
Yes	1.67 (1.25–2.23)	<0.001	1.40 (1.04–1.89)	0.027

Ref, reference; CI, confidence interval; AKI, acute kidney injury; TBI, traumatic brain injury; ICU, intensive care unit.

Model 1: the crude model.

Model 2: adjusted for SAPS II, calcium, mechanical ventilation, vasopressors, and platelet transfusion.

### Subgroup analysis

As shown in Table 3, further stratified analysis revealed that the association between early coagulation disorders and severe AKI in TBI patients remained strong, after adjusting for all covariates. Compared to the patients without early coagulation disorders, those with early coagulation disorders had an increased risk of AKI. Specifically, among patients older than 65 years, the risk of AKI increased by 91% (HR = 1.46, 95%CI: 1.01–2.14). In patients with GCS scores ≤13 (HR = 1.91, 95%CI: 1.16–3.15), the risk of AKI increased by 91%, with all *p*-values less than 0.05.

**Table 3:** Association between early coagulation disorders and the risk of AKI in the patients with TBI admitted to the ICU based on age and the GCS score.

Variates	Model l				Model 2			
	Age < 65		Age ≥ 65		Age < 65		Age ≥ 65	
	HR (95%CI)	*p*	HR (95%CI)	*p*	HR (95%CI)	*p*	HR (95%CI)	*p*
Early coagulation disorder								
No	Ref		Ref		Ref		Ref	
Yes	1.81 (1.14–2.87)	0.013	1.58 (1.09–2.28)	0.016	1.15 (0.69–1.94)	0.590	1.46 (1.01–2.14)	0.048

Variates	GCS: ≤13		GCS: 14–15		GCS: ≤13		GCS: 14–15	
	HR (95%CI)	*p*	HR (95%CI)	*p*	HR (95%CI)	*p*	HR (95%CI)	*p*
Early coagulation disorder								
No	Ref		Ref		Ref		Ref	
Yes	2.39 (1.47–3.89)	<0.001	1.36 (0.95–1.96)	0.097	1.91 (1.16–3.15)	0.012	1.16 (0.79–1.70)	0.440

Ref, reference; CI, confidence interval; AKI, acute kidney injury; TBI, traumatic brain injury; ICU, intensive care unit; GCS, Glasgow Coma Scale.

Model 1: the crude model.

Model 2: adjusted for SAPS II, calcium, mechanical ventilation, vasopressors, and platelet transfusion.

## Discussion

The results of the present study indicated that early coagulation disorders were positively associated with a higher risk of severe AKI in TBI patients admitted to the ICU, especially among those aged ≥65 years and those with GCS scores of ≤13. It is of great significance to monitor the coagulation biomarker levels in TBI patients and identify the high-risk population susceptible to AKI to improve the prognosis of TBI patients.

In this study, early coagulation disorders were significantly correlated with a higher risk of severe AKI in TBI patients admitted to the ICU compared to TBI patients without early coagulation disorders. Several previous studies have focused on the relationship between coagulation function and kidney injury (8, 11). A retrospective, observational study on patients with septic shock suggested that the APTT, prothrombin time (PT), and D-dimer level upon admission to the ICU were significant risk factors for AKI. Another study by Katamama et al. (16), which focused on the same population, aimed to explore the interactive connection between sepsis-induced AKI and several biomarkers of endothelial injury and the coagulation system. The results of their study reported that PT and PLT levels were associated with the development of AKI in patients with sepsis. Benediktsson et al. (17) reported that prolonged APTT and PT at the time of ICU admission were associated with increased mortality in patients with sepsis. The aforementioned studies differed from our study in the selection of the population and coagulation function biomarkers, but they all concluded that coagulation dysfunction is associated with an increased risk of AKI. The main pathogenesis of kidney injury was reported to be immune-inflammatory injury and metabolic abnormalities, accompanied by abnormal platelet activation and coagulation/fibrinolysis balance disorders (18). Watanabe M et al. reported that a hypercoagulable state was an important factor in the pathogenesis of AKI (19). The tissue factor/factor VIIa complex and factor Xa in the coagulation cascade activate protease-activated receptor 2 (PAR2), and PAR2-mediated inflammation exacerbates kidney injury in models of diabetic nephropathy and adenine-induced renal fibrosis (20).

TBI was related to an increased risk of coagulopathy (21). The present study indicated that TBI was associated with early coagulation disorders, including a decrease in platelet count and lengthening of clotting time. A retrospective, observational study by Yuan et al. (8) reported that 18.6% of the study population developed coagulopathy after isolated TBI (iTBI) and that 30.4% of patients with severe iTBI experienced coagulopathy. Early coagulopathy was associated with higher in-hospital mortality, with the INR >1.25 and APTT >36 s. The initial injury of TBI often involves disruptions of the cerebral vasculature and/or pathological alterations of the blood–brain barrier, which can evolve into hemorrhagic lesions. Then, factors associated with TBI can alter the body’s hemocoagulative state and disrupt the delicate balance between bleeding and thrombosis formation, resulting in coagulopathy and a significant exacerbation of the initial injury (22, 23). Coagulation may be amenable to treatment, and adequate and prompt interventions can prevent poorer outcomes of TBI.

The GCS score is mainly used to assess the severity of craniocerebral injury (24). In the subgroup analyses of the present study, early coagulation disorders were associated with a higher risk of severe AKI in TBI patients with GCS score of ≤13 (representing moderate-to-severe brain injury). Patients with moderate-to-severe TBI have a worse prognosis. Monitoring the coagulation function of these patients and taking appropriate treatment measures on time play an important role in reducing the prognostic burden. Moreover, for TBI patients aged over 65 years, attention to changes in the INR, PLT, and APTT may help decrease the risk of severe AKI. TBI is a significant problem in older adults. In individuals aged 65 years and older, TBI was reported to be responsible for more than 80,000 emergency department visits each year (25). Previous studies have shown clear differences in the coagulation process between the elderly and the young (26). With aging, levels of fibrinogen and coagulation proteins in plasma increase, which promotes the occurrence of thrombotic events. Paying attention to the health of the coagulation system in elderly patients with TBI will benefit their prognosis.

Currently, few studies have focused on the association between early coagulopathy and the risk of severe AKI in TBI patients admitted to the ICU. Our findings have several implications for clinicians. First, early coagulation disorders should be considered, in part, a clue to the risk of AKI in TBI patients. Second, monitoring the levels of coagulation biomarkers and kidney function in TBI patients and identifying the high-risk population for AKI on time are beneficial for improving the prognosis of TBI patients. Although the use of a large dataset from the public MIMIC-IV database provided substantial support for our results, our study still has several limitations. First, as a single-center retrospective study, despite conducting strong statistical corrections, the limited sample size and data bias were inevitable. It was not possible to clarify the association between early coagulation disorders and the risk of severe AKI in TBI patients, as is done in prospective studies, so our study lacks some persuasive power to that extent. Second, our study only focused on TBI patients admitted to the ICU, and therefore, the associations between early coagulopathy and the risk of severe AKI in TBI patients admitted to the general ward need to be investigated in the future. Third, owing to the limitations of the MIMIC database, some characteristics of TBI and AKI were not extracted, so we were unable to include all the covariates affecting the study outcomes. However, a retrospective study involving these patients makes it difficult to draw robust conclusions. Therefore, more prospective, multicenter trials to evaluate the relationship between early coagulation disorders and severe AKI in TBI patients are necessary.

## Conclusion

In the present study, we found that early coagulation disorders were an important risk factor for severe AKI in the patients with TBI admitted to the ICU. Monitoring the early coagulation function in TBI patients and implementing necessary interventions are conducive to the early identification of severe AKI, significantly reducing the disease burden associated with TBI.

## Data Availability

Publicly available datasets were analyzed in this study. This data can be found here: MIMIC-IV database, https://mimic.physionet.org/iv.

## Footnotes

^1^ http://physionet.org/content/mimiciv/1.0/
