# Supplementary material for: Association between early coagulation disorders and the risk of severe acute kidney injury in traumatic brain injury patients: a retrospective cohort study using the MIMIC-IV database
Source: Front Neurol. 2025 Feb 18;15:1407107. doi: 10.3389/fneur.2024.1407107 (PMC11876056; doi:10.3389/fneur.2024.1407107)
Supplement: Supplementary file 1 [file Table_1.docx]

Table S1 Univariate cox proportional hazards models of sever AKI in TBI patients admitted to the ICU

| Variates | HR (95%CI) | *P* |
| --- | --- | --- |
| Age | 1.01 (1.00-1.01) | 0.146 |
| Gender |  |  |
| Female | Ref |  |
| Male | 1.06 (0.78-1.42) | 0.720 |
| Race |  |  |
| White | Ref |  |
| Black | 1.11 (0.63-1.97) | 0.722 |
| Other | 0.89 (0.65-1.23) | 0.488 |
| Insurance |  |  |
| Medicaid | Ref |  |
| Medicare | 1.21 (0.67-2.16) | 0.524 |
| Other | 1.00 (0.56-1.79) | 0.994 |
| Marital status |  |  |
| Married | Ref |  |
| Spinsterhood | 0.84 (0.61-1.15) | 0.279 |
| ICU type |  |  |
| SICU | Ref |  |
| TSICU | 0.90 (0.63-1.29) | 0.583 |
| Other | 0.88 (0.61-1.26) | 0.479 |
| 24 h urine output | 1.00 (1.00-1.00) | 0.977 |
| Heart rate | 1.01 (1.00-1.01) | 0.210 |
| Systolic | 1.00 (1.00-1.01) | 0.226 |
| Diastolic | 1.00 (0.99-1.01) | 0.993 |
| Respiratory rate | 1.00 (0.98-1.03) | 0.736 |
| Temperature | 0.84 (0.70-1.02) | 0.087 |
| Spo2 | 1.02 (0.97-1.08) | 0.378 |
| SAPSII | 1.02 (1.01-1.04) | <.001 |
| GCS | 0.98 (0.93-1.04) | 0.466 |
| GCS |  |  |
| ≤13 | Ref |  |
| 14-15 | 1.22 (0.91-1.64) | 0.187 |
| CCI | 1.06 (0.99-1.13) | 0.095 |
| WBC | 1.00 (0.98-1.02) | 0.973 |
| Hematocrit | 0.98 (0.96-1.01) | 0.157 |
| Hemoglobin | 0.96 (0.89-1.03) | 0.279 |
| RDW | 1.07 (1.00-1.15) | 0.053 |
| BUN | 1.01 (0.99-1.02) | 0.321 |
| Glucose | 1.00 (1.00-1.00) | 0.554 |
| Calcium | 0.92 (0.87-0.96) | <.001 |
| Sodium | 0.98 (0.95-1.00) | 0.076 |
| Potassium | 0.85 (0.66-1.08) | 0.183 |
| Chloride | 1.00 (0.97-1.02) | 0.744 |
| Bicarbonate | 1.01 (0.97-1.05) | 0.692 |
| Anion gap | 0.98 (0.94-1.02) | 0.280 |
| Mechanical ventilation |  |  |
| No | Ref |  |
| Yes | 1.69 (1.23-2.32) | 0.001 |
| Vasopressors |  |  |
| No | Ref |  |
| Yes | 1.87 (1.27-2.76) | 0.002 |
| Platelet transfusion |  |  |
| No | Ref |  |
| Yes | 1.90 (1.33-2.73) | <.001 |
| Mannitol |  |  |
| No | Ref |  |
| Yes | 1.97 (0.93-4.20) | 0.078 |
| Neurosurgical intervention |  |  |
| No | Ref |  |
| Yes | 1.24 (0.31-5.01) | 0.758 |
| Diuretic |  |  |
| No | Ref |  |
| Yes | 1.70 (0.97-2.98) | 0.066 |
| Vitamin k |  |  |
| No | Ref |  |
| Yes | 1.23 (0.51-3.00) | 0.645 |

HR: hazard ratio: CI: confidence interval; AKI: acute kidney injury; TBI: traumatic brain injury; SICU: Surgical Intensive Care Unit; TSICU: Trauma SICU; SAPSII: Simplified acute physiology score II; GCS: Glasgow Coma Scale; CCI: Charlson Comorbidity Index; WBC: white blood cell count; RDW: red blood cell distribution width; BUN: blood urea nitrogen.

Table S2 Sensitivity analyses before and after imputation

| Variables | Total (n=1,692) | After imputation (n=846) | Before imputation (n=846) | Statistics | *P* |
| --- | --- | --- | --- | --- | --- |
| Marital status, n (%) |  |  |  | χ^2^=0.549 | 0.459 |
| Married | 1070 (68.41) | 572 (67.61) | 498 (69.36) |  |  |
| Spinsterhood | 494 (31.59) | 274 (32.39) | 220 (30.64) |  |  |
| Systolic, Mean ±SD | 133.04 ± 21.80 | 133.04 ± 21.80 | 133.04 ± 21.81 | t=-0.01 | 0.993 |
| Diastolic, Mean ±SD | 72.92 ± 16.69 | 72.91 ± 16.69 | 72.93 ± 16.69 | t=-0.03 | 0.976 |
| Respiratory rate, Mean ±SD | 18.38 ± 5.02 | 18.38 ± 5.01 | 18.37 ± 5.04 | t=0.05 | 0.960 |
| Temperature, Mean ±SD | 36.84 ± 0.70 | 36.84 ± 0.70 | 36.84 ± 0.70 | t=0.01 | 0.995 |
| BUN, M (Q_1_,Q_3_) | 15.00 (11.00, 20.00) | 15.00 (11.00, 20.00) | 15.00 (11.00, 20.00) | Z=-0.004 | 0.997 |
| Glucose, M (Q_1_,Q_3_) | 119.00 (101.00, 147.00) | 119.50 (101.00, 147.00) | 119.00 (101.00, 147.00) | Z=-0.018 | 0.986 |
| Calcium, Mean ±SD | 7.85 ± 2.33 | 7.86 ± 2.32 | 7.84 ± 2.34 | t=0.13 | 0.898 |
| Sodium, Mean ±SD | 139.02 ± 4.79 | 139.02 ± 4.79 | 139.02 ± 4.79 | t=0.02 | 0.984 |
| Potassium, Mean ±SD | 4.03 ± 0.64 | 4.03 ± 0.64 | 4.03 ± 0.64 | t=-0.00 | 0.999 |
| Chloride, Mean ±SD | 103.66 ± 5.41 | 103.66 ± 5.41 | 103.66 ± 5.41 | t=0.00 | 0.999 |
| Bicarbonate, Mean ±SD | 23.33 ± 3.63 | 23.33 ± 3.63 | 23.32 ± 3.63 | t=0.02 | 0.982 |
| Anion gap, Mean ±SD | 15.00 ± 3.53 | 15.00 ± 3.53 | 14.99 ± 3.53 | t=0.03 | 0.978 |
| 24 h urine output, M(Q_1_,Q_3_) | 1942.00 (1350.00, 2649.50) | 1942.00 (1350.00, 2649.00) | 1941.00 (1355.00, 2650.00) | Z=0.106 | 0.915 |

BUN: blood urea nitrogen; SD: standard deviation, M: median, Q_1_:1st quartile, Q_3_:3st quartile.
